# Induction of autophagy improves skin and hair conditions in dogs with underlying diseases

**DOI:** 10.3389/fvets.2023.1078259

**Published:** 2023-01-26

**Authors:** Yoonji Kim, Seung-Hwa Lee, Yunji Song, Sekyoo Jeong, Ha-Jung Kim

**Affiliations:** ^1^Department of Internal Medicine, College of Veterinary Medicine, Chonnam National University, Gwangju, Republic of Korea; ^2^BK 21 Project Team, College of Veterinary Medicine, Chonnam National University, Gwangju, Republic of Korea; ^3^Asan Institute for Life Sciences, University of Ulsan College of Medicine, Seoul, Republic of Korea; ^4^Research Team, Incospharm Corp., Daejeon, Republic of Korea

**Keywords:** autophagy, dog, skin, keratinocytes, hair, TEWL

## Abstract

**Background:**

Autophagy was reported to play a crucial role in maintaining general and skin health.

**Methods:**

The study used a synthesized autophagy inducer (AI) (Aquatide™ cospharm Inc.; Daejeon, Korea), for evaluating the effects of autophagy on skin and hair in dogs. Twenty-two dogs with poor skin and hair which were diagnosed with canine atopic dermatitis (CAD) or pituitary-dependent hyperadrenocorticism (PDH) were included. Clinical scores using Canine Atopic Dermatitis Extent and Severity Index-04 (CADESI-04), Pruritus Visual Analog Scale (PVAS) and skin barrier function using measurement of transepidermal water loss (TEWL) were evaluated and canine keratinocytes were also used *in vitro* investigation of pro-inflammatory cytokines after AI treatment.

**Results:**

In the AI group, clinical scores and skin barrier function were improved at week 8 significantly compared to in the other groups. In particular, the AI significantly improved the hair surface damage at 8 weeks compared to the baseline. *In vitro*, the AI reduced pro-inflammatory cytokines by activating the 78-kDa glucose-regulated protein (GRP78).

**Conclusion:**

AI improve skin barrier function and hair damage and reduce pro-inflammatory cytokines by inhibiting reactive oxygen species (ROS) production in dogs.

## 1. Introduction

The canine skin and hair coat are important predictors of underlying systemic diseases and the general health status. Symptoms of poor skin and hair conditions in dogs are very broad, but commonly include localized or extensive itching, dryness, hair loss, and other unusual behaviors such as excessive licking and scratching (1, 2). Diseases with bad effects on the dogs' skin and hair representatively include many conditions like atopic dermatitis, hyperadrenocorticism, and malignant tumors (3–5). Therapeutic options for these diseases vary; canine atopic dermatitis (CAD) can be treated with anti-inflammatory drugs, antipruritic drugs, and allergen-specific immunotherapy (6), hyperadrenocorticism is treated with trilostane (7), and anti-cancer drugs like toceranib, lomustine, and vincristine are used in tumors (8, 9). However, the efficacy of current therapeutic options could be limited in some cases of CAD and additional treatments might be needed (10).

Autophagy is central to skin health and plays a critical role in controlling skin aging, inflammation, and immune responses (11–13). Under external (e.g., UV irradiation) and internal (e.g., stress) conditions, induced-autophagy removes aged subcellular organelles and proteins while it also regulates skin homeostasis, functions of keratinocytes, proliferation, and differentiation of epidermal stem cells to protect skin health (13–15). In addition, autophagy can influence hair development by improving the period of the hair cycle, shortening the period between the telogen phase to the anagen phase, and inducing hair regeneration (13, 16). Therefore, maintenance of appropriate autophagy is critical for host skin homeostasis, and modulation of the autophagy and function is a promising therapeutic strategy. However, the studies of mechanism and clinical effects of autophagy on the skin health are not enough.

Heptasodium hexacarboxymethyl dipeptide-12 is a recently synthesized autophagy inducer branded as Aquatide™ (Incospharm Incorporation, Dajeon, Korea) (17). Aquatide™ also contains Resveratrol, which activates sirtuin-1, and pyrrolidone carboxylic acid, which is a skin moisturizing ingredient (17–19). Recent evidence indicates that Aquatide™ can exert the anti-oxidant and anti-aging effects in humans by activating autophagy through direct binding onto sirtuin-1 (20).

In the present study, we firstly investigated the effects of autophagy induction on the skin and hair conditions topically in dogs and it may be a useful model for investigations of autophagy in human.

## 2. Materials and methods

### 2.1. Materials

Synthesized Aquatide™ (Incospharm Inc., Daejeon, Korea) was used for evaluating the effects of autophagy inducer on dogs' skin and hair. In a prior study, safety assessments were completed in Beagles and the research also complied with the Institutional Animal Care and Use Committee guidelines at Chonnam National University (identification code No. CNU IACUC-YB-2019-26).

Two types of topical formulation were prepared for the study; the test products contained 2% Aquatide™, glycerin, propylene glycol, and 1,3-butylene glycol and the vehicle products contained the same components without Aquatide™. The topical formulation was applied twice a day to the whole body for 8 weeks.

### 2.2. Study population

A total of 31 client-owned dogs who visited the veterinary teaching hospital were included. All the owners approved the study and signed an informed consent. Twenty-two patient dogs with poor skin and hair conditions based on physical examination were included regardless of the underlying diseases. The average range of their age was 7.79 ± 3.62 years. They consisted of three females, 16 spayed females, one male, and 13 neutered males. The patients included six Poodles, six Pomeranian, six Malteses, four Mixed, two Spitzs, two Cocker Spaniels, one Chihuahua, one French Bulldog, one Bichon Frise, one Shih tzu, one Jindo, one Miniature Pinscher and one Siberian Huskey. Healthy control group consisted of nine dogs with the average range of age of 6.00 ± 3.40 years. They consisted of two females, three spayed females, and four neutered males. The healthy conrols included three Poodles, one Siberian Huskey, one Cocker Spaniel, one Spitz, one miniature Pinscher, and two mixed breeds.

### 2.3. Treatment group

The enrolled dogs were maintained for the treatment for their underlying diseases during the study 90 (for 8 weeks). For example, CAD patients were treated with anti-inflammatory drugs (e.g., cyclosporine (Cipol-N^®^ oral solution, CKD Pharm, Seoul, Korea) and hypoallergenic diets (e.g., hypoallergenic, Royal Canine, Dissay, France) and PDH patients were prescribed trilostane (Vetoryl^®^, Dechra, Northwich, United Kingdom).

The dogs were divided into 3 groups in a randomized and double-blinded manner. Staff not involved in the treatment randomly allocated the dogs into three groups using a random number table procedure. Five dogs with CAD (age 6.60 ± 3.07 years) and two dogs with PDH (age 13 ± 1 years) were applied the Aquatide™ product. Three dogs with CAD (age 6.33 ± 4.11 years) and three dogs with PDH (age 10.33 ± 3.30 years) were applied the vehicle product. Other seven dogs with CAD (age 7.43 ± 2.13 years) and two dogs with PDH (age 7.50 ± 2.50 years) were not applied with any topical product. Nine client-owned dogs were recruited as the healthy control (age 6.00 ± 3.39 years). These dogs were determined to be healthy based upon medical history and physical examination. To assess the skin and hair conditions, skin examinations and hair damage in healthy controls at week 0 and in experimental groups at weeks 0, 4, and 8 were evaluated. As for methods used for diagnosis, Favrot's criteria was used in CAD. Adrenocorticotropic hormone stimulation test and high-dose dexamethasone suppression test was used in PDH.

### 2.4. Skin examination

#### 2.4.1. Evaluation of skin lesions

Not only atopic dermatitis, but also all the patients were evaluated using Canine Atopic Dermatitis Extent and Severity Index-04 (CADESI-04) (3) for skin lesion scoring.

#### 2.4.2. Measurement of transepidermal water loss

TEWL was measured through the closed-chamber method using GPSkin Barrier^®^ (GPOWER Inc., Seoul, Korea) (21–23). The assessment regions, including the left and right sides of the concave surface of the pinna, axilla, and inguinal area were repeatedly measured. All sites were measured three times by placing the probe in the same location. All the TEWL values were measured after stabilizing the dogs for 30 min in a controlled room at room temperature, with temperature of 26 ± 1°C and relative humidity of 50 ± 5%.

#### 2.4.3. Clinical assessment of pruritus

For collective evaluation of skin condition, Pruritus Visual Analog Scale (PVAS) was graded for the assessment of itching (24). The scores ranged from 0 to 10.

### 2.5. Evaluation of hair condition based on hair damage

The collected hair samples from flank including hair bulb were stored at room temperature until analysis. Grades of hair damage include hair surface damage, hair cuticle layers damage, and hair cortex damage (25). The hair surface damage was evaluated using scanning electron microscopy (EM-30, 30 kV; COXEM, Daejeon, Korea). Each hair was photographed three times and evaluated by two investigators. The score of hair surface damage is 0 to 4.

### 2.6. Canine keratinocytes experiments

#### 2.6.1. Cell culture and Aquatide™ treatment

Canine epidermal keratinocytes were purchased from the CELLnTEC and were maintained at 37°C in a humidified incubator at 5% CO_2_ in CnT-09 culture medium. For the experiment, Canine keratinocytes (5 × 10^5^) were seeded in a 60 mm cell culture dish, incubated for 24 h, and pre-treated with Aquatide™ (1, 10, 100 μg/mL) or rapamycin (5 μM) for 1 h. The dose of Aquatide™ was determined based on a prior study (17, 20). After removing the treatment media, cells were washed with PBS and cultured in media with lipopolysaccharide (LPS) (10 μg/mL) for 4 h. Then, the cells were washed twice with cold PBS and harvested. MTT test was performed to check cell viability.

#### 2.6.2. Western blot and real-time RT-PCR

Realtime PCR was performed with TaqMan Gene Expression Assays from Life Technologies (GRP78, HS00946086_g1; GapdH, Cf04419463_gH). The protein levels of Glucose regulated protein (GRP-78), Interleukin (IL) 4 and IL-13 in keratinocyte were measured using western blotting analysis. The relative expression of GRP-78 was measured using qPCR. Each signal was normalized against GAPDH signals in the same sample. Details are provided in the methods of the Supplementary material.

### 2.7. Statistical analysis

All the statistical analyses were performed using GraphPad Prism version 9 (GraphPad Software, La Jolla, CA, USA). The Shapiro-Wilk tests were performed to analyze the normality of all data. The unpaired *T*-test was used to compare skin condition between healthy control and patient group at the starting point of the trial. Other data was analyzed using ANOVA with subsequent Tukey's multiple comparison tests to establish the difference between groups. Mean ± standard deviation (SD) was used to describe the distribution of continuous variables. Statistical significance was set at *P* < 0.05.

## 3. Results

### 3.1. Subject characteristics

**Figure 1:**
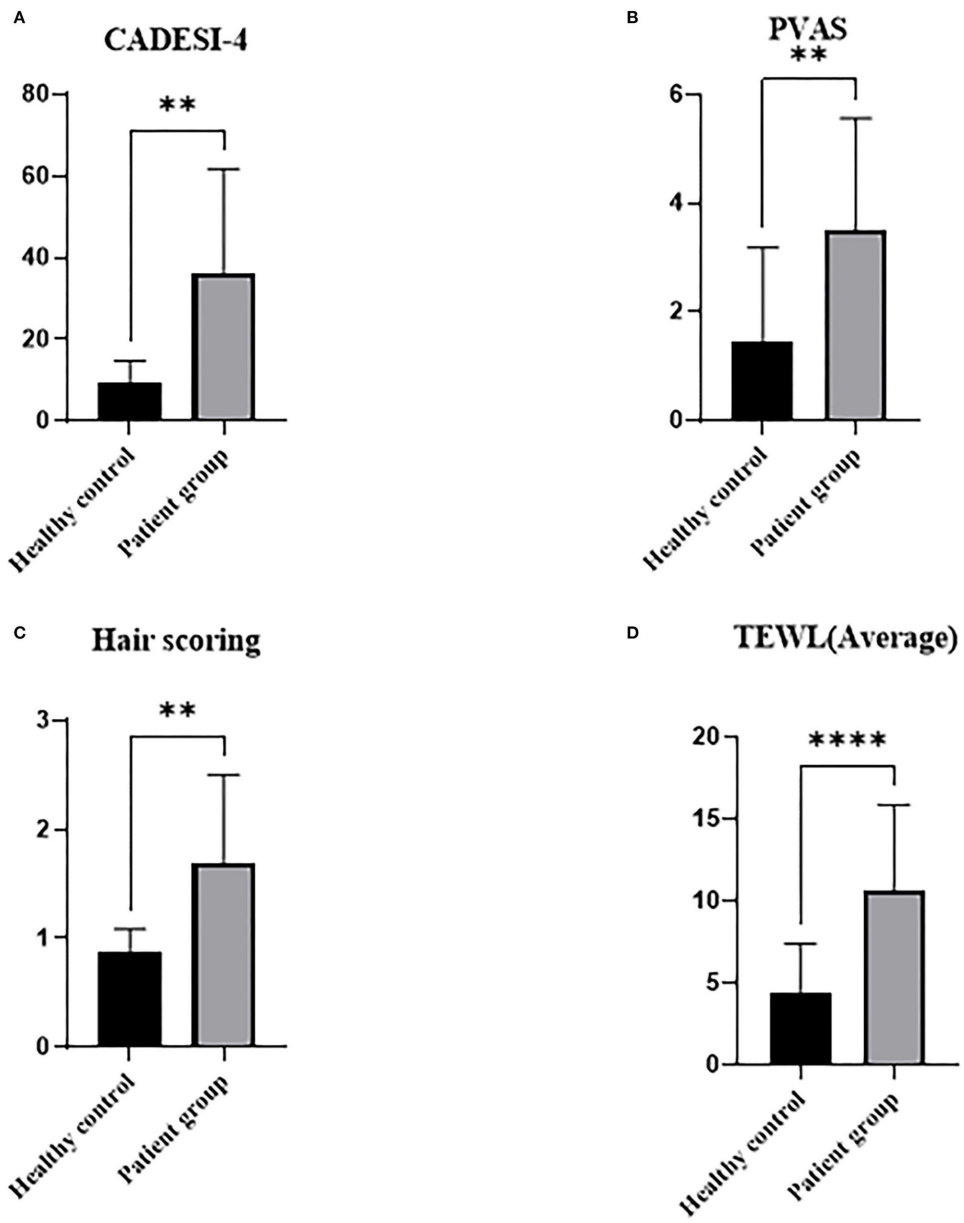
Graphs of skin and hair conditions by underlying diseases in dogs at starting point. Healthy controls were determined based upon medical history and physical examination. Treatment group includes CAD and PDH. CAD, canine atopic dermatitis; CADESI-04, canine atopic dermatitis extent and severity index-04; PDH, pituitary dependent hyperadrenocorticism; PVAS, pruritus visual analog scale; TEWL, transepidermal water loss. **p < 0.01, ****p < 0.0001.

The demographics of the total study population at the starting point of the trial are shown in Figure 1. Among these experimental groups, diseases of patients are presented in Table 1. CADESI-04, PVAS, and hair scoring was significantly higher in the treatment group (CAD + PDH) than the healthy controls (*P* < 0.01). TEWL was statistically higher in the treatment group than in the control group (*P* < 0.0001).

**Table 1:** Categorized illnesses in 22 dogs.

**Diagnosis**	***n***	**%**
CAD	15	68.2
PDH	7	31.8
Total	22	100

CAD, canine atopic dermatitis; PDH, pituitary dependent hyperadrenocorticism.

### 3.2. Induction of autophagy improves skin conditions and hair quality in dogs with underlying diseases

**Figure 2:**
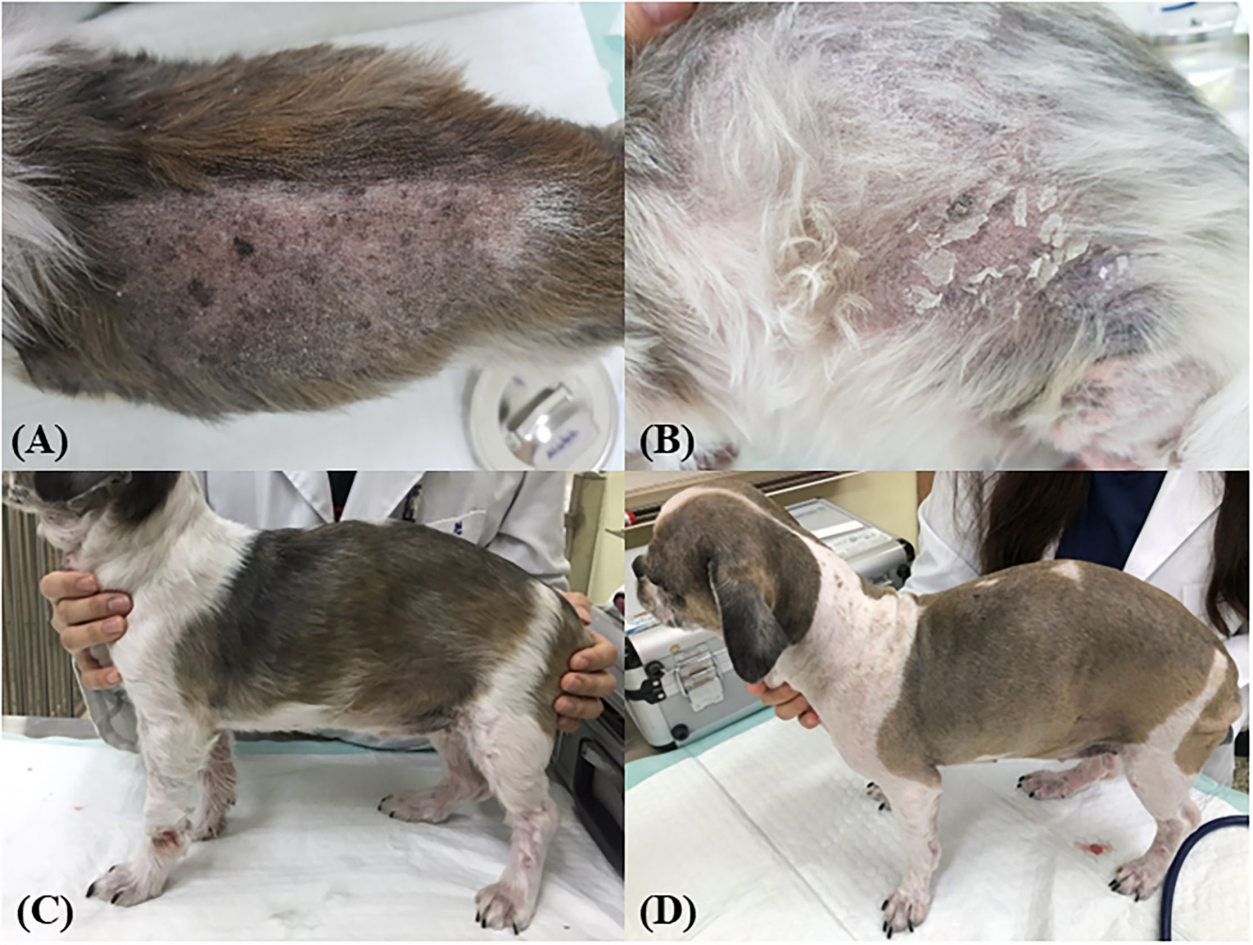
Induction of autophagy improves skin lesions and hair quality in dogs with CAD. The alopecia on the left flank and many keratins were identified on the back and left flank at week 0 **(A, B)**. After 4 weeks, the lesions and the hair quality were improved **(C)**. At week 8, improved lesions were maintained **(D)**.

**Figure 3:**
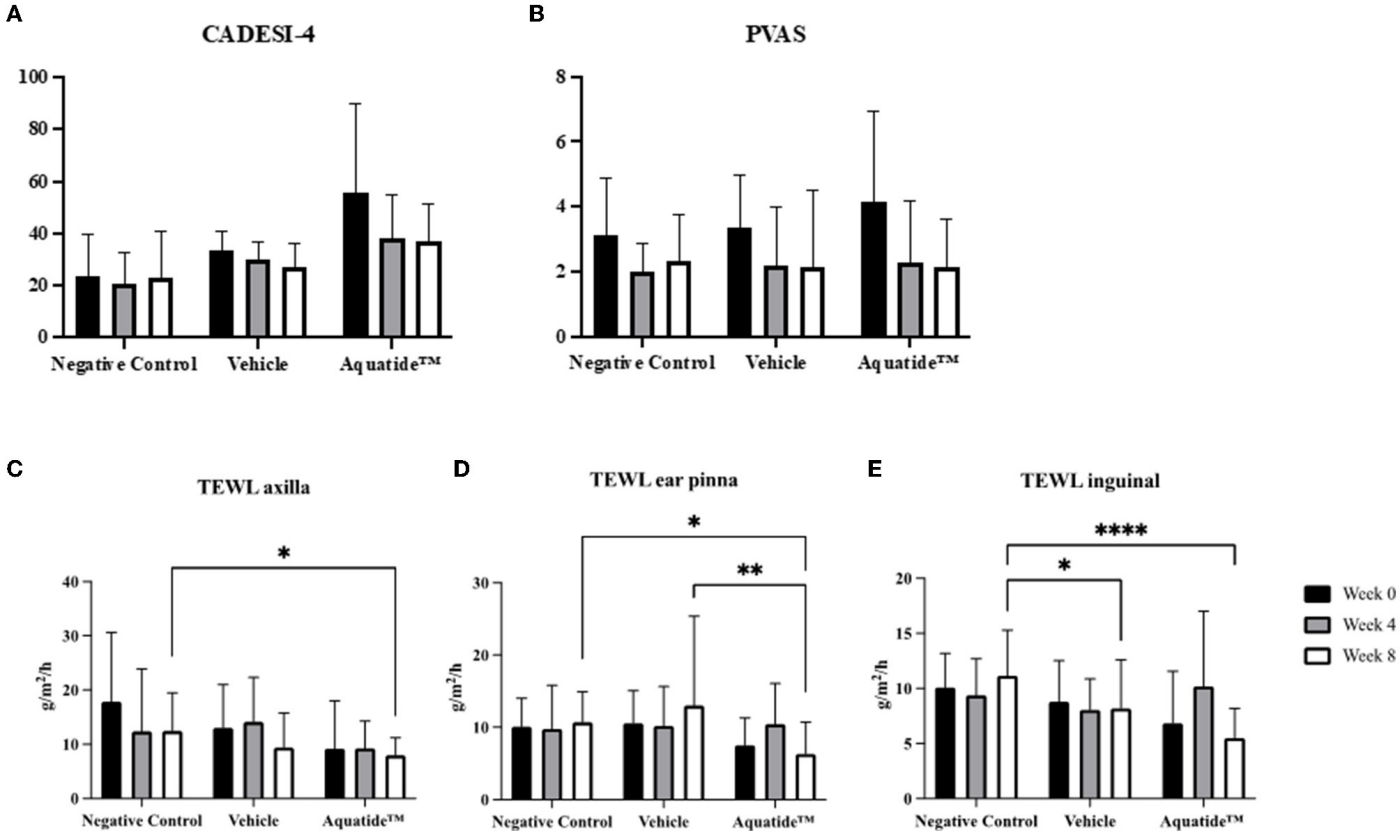
Graphs of CADESI-4, PVAS and TEWL at week 0, 4, and 8 in the treatment group. Induction of autophagy significantly reduces TEWL in dogs with underlying diseases. CADESI-04, canine atopic dermatitis extent and severity index-04; VAS, visual analog scale; TEWL, transepidermal water loss. *p < 0.05, **p < 0.01, and ****p < 0.0001.

**Figure 4:**
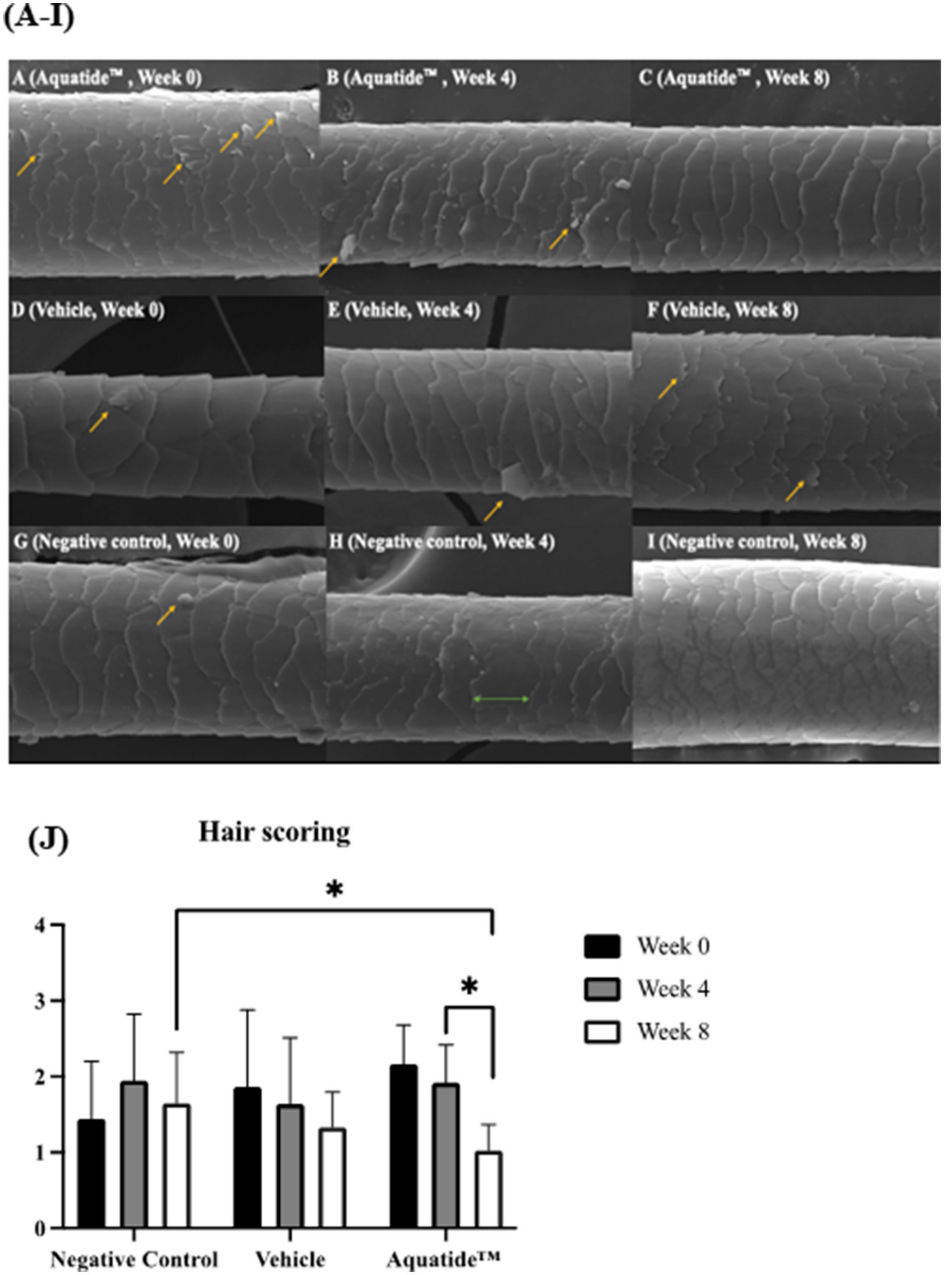
Induction of autophagy improves hair surface damage and hair cuticle layers in dogs with underlying diseases. **(A–I)** Scanning electron microscopic photographs for evaluating hair surface damage. The arrows points to the hair surface damage. It was confirmed that the hair surface was improved at week 8 after Aquatide™ was applied. **(J)** Hair scoring in the treatment group. When Aquatide™ was applied, hair scoring was significantly decreased at week 8 compared to week 0 and week 4, and was significantly lower than in the negative control. *p < 0.05.

To investigate whether autophagy induction has the potential to alleviate skin conditions, we applied the Aquatide™ topical formulation twice a day to the whole body for 8 weeks. In continuous visual evaluations, the dogs who were applied Aquatide^TM^ showed improved skin lesions and hair conditions after the 8 weeks compared to week 0 (Figure 2). In addition, the dogs who were applied Aquatide™ showed significantly decreased TEWL in the skin than the negative controls (axilla, *P* < 0.05; ear pinna, *P* < 0.05; inguinal regions, *P* < 0.0001; Figures 3C–E). The dogs in the Aquatide™ group also had a lower CADESI-4 and PVAS compared to the vehicle group, although there was no significant difference (Figures 3A, B). In assessments of the hair conditions in these dogs, Aquatide™ treatment reduced hair surface damage and hair cuticle layers compared to the negative and vehicle control groups (*P* < 0.05; Figure 4). These findings indicate that Aquatide™ treatment effectively alleviated skin conditions and hair quality in dogs with underlying diseases.

### 3.3. Preconditioning-induced autophagy reduces pro-inflammatory cytokines through GRP78 activation in canine keratinocyte cells

**Figure 5:**
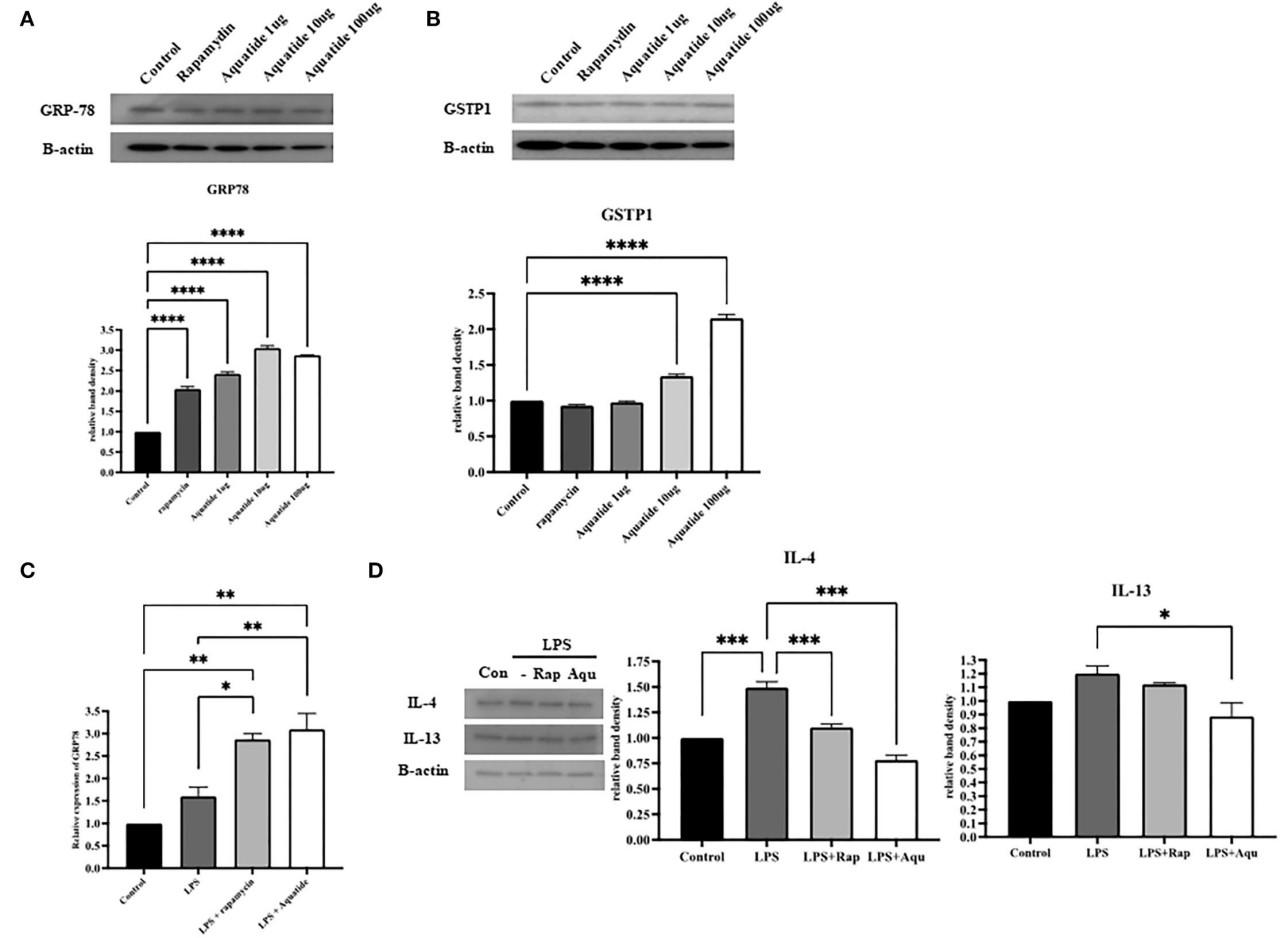
Preconditioning-induced autophagy reduces pro-inflammatory cytokines through GRP78 activation in canine keratinocyte cells. The antioxidant-related protein levels of **(A)** GRP-78 and **(B)** GSTP1 and mRNA levels of **(C)** GRP-78 were assessed by western blot and real-time PCR. **(D)** pro-inflammatory cytokines (IL-4 and IL-13) were assessed by western blot analysis. Statistical significance was determined using ANOVA and Tukey's multiple comparison tests. *P < 0.05, **P < 0.01, ***P < 0.001, and ****P < 0.0001.

To confirm if activation of autophagy regulates oxidative stress, we examined the levels of GRP-78 after Aquatide™ treatment *in vitro*. As shown in Figures 5A, B, the GRP-78 protein level in the Aquatide™ group gradually increased in a dose-dependent manner than those of the control and cells incubated with rapamycin (positive control group). Next, we examined whether Aquaitde-induced activation alters pro-inflammatory cytokine levels after LPS stimulation. As shown in Figure 5C, D, pretreatment of Aquatide™ significantly lowered pro-inflammatory cytokines (IL-4 and IL-13) in keratinocytes, compared with non-treated cells after LPS stimulation. In addition, pretreatment with Aquatide™ significantly increased GRP-78 gene expression after LPS stimulation. These findings indicate that pretreatment with Aquatide™ effectively decreases pro-inflammatory cytokines in the keratinocytes by activating GRP-78 *in vitro*.

## 4. Discussion

Here, we demonstrated that topical Aquatide™ treatment in dogs with skin disease alleviated skin and hair conditions. In addition, preconditioning-induced autophagy decreased pro-inflammatory cytokines and increased GRP-78 protein levels after LPS stimulation in the keratinocyte cells. Our results indicated that increased autophagy activation ameliorates skin and hair conditions *via* the modulation of oxidative stress-mediated inflammation in the skin of canine skin disorder.

Previous studies have reported that an imbalance of skin homeostasis from underlying diseases contributes to the development of skin and hair disorders (26–28). In this study, the dogs enrolled had underlying diseases that affect skin health, such as CAD and PDH. Those canine primary diseases may show poor skin conditions, such as pruritic inflammation in CAD (3). Alopecia and seborrhea sicca can be seen in hyperadrenocorticism (4).

Application of Aquatide™ improved skin clinical features, which were considered typical abnormalities induced by underlying diseases. Previously, some studies reported that the induction of autophagy has important anti-oxidant effects with preventive properties in skin disorders by reducing inflammation (29, 30). In addition, autophagy activation is reported to provide an anti-inflammatory promoting activity in the mouse skin (31). Therefore, Aquatide™ application might have exerted improved effects against skin disorders by blocking inflammatory-related responses, especially with anti-oxidation through induction of autophagy.

In this study, it was found that the effect of improved hair quality was greater than that of clinical scores such as CADESI-04 and PVAS in dogs. *In vitro*, anti-inflammatory effects were observed, but no statistical significance was observed in CADESI-04 and PVAS, which are related to clinical symptoms. The discrepancy in the outcomes between the clinical and preclinical experiments may be due to not effective concentration of AI on the skin or some different environmental factor in skin *in vivo* and cells *in vitro*.

Chronic skin inflammation is associated with the overproduction of reactive oxygen species (ROS), defined as oxidative stress (32). Excessive ROS can directly cause damage to the cells, and eventually exceeds the defense capacity of the antioxidant system, leading to the pathogenesis of AD, as well as cutaneous diseases (33–35). Autophagy is an evolutionarily conserved homeostatic cellular process to promote cell survival and adaptive responses during oxidative and/or genotoxic stress conditions (36, 37).

Previous studies have reported that autophagy inducers such as rapamycin and Aquatide™ provide optimal protection against skin inflammation and cellular senescence *via* suppressing the production of ROS and the maintenance of cellular homeostasis (17, 20, 31, 38). In addition, as an autophagy inducer, rapamycin alleviated oxidative stress-related ER stress in cellular-protection *via* the promotion of GRP78/mTOR signaling pathway (39). ROS causes death of hair cells, but autophagy can decrease ROS levels (40, 41). Therefore, the hair quality improvement effects could be also attributed to the ROS-related mechanism.

In addition, the expression of IL-4 and IL-13, which are pro-inflammatory cytokines associated with AD, was decreased while the expression of GRP-78 was increased in the keratinocyte cells after Aquatide™ treatment. These results suggest that induction of autophagy promotes GRP78-related function directly or indirectly, and therefore may suppress inflammation in the skin.

Our present study has some limitations. Autophagy triggers hair regeneration (16), but we could not evaluate the effect of hair cycle improvement in dogs. It was not easy to evaluate the effect of hair regeneration in this study because the evaluation period was short, and many dogs had been hair clipping, instead, we evaluated the hair quality improvements effects through scanning electron microscopy. Importantly, to date, a direct relationship between Aquatide™ and anti-oxidant in canines has not been identified. Further studies are required to confirm this relationship and elucidate the underlying mechanism. Finally, in the Aquatide™ group, CADESI-04 and PVAS decreased, but there were significant differences because the number of dogs in this study was relatively small. Further studies should overcome this limitation. Nevertheless, this is the first study to evaluate the effects of a topical formulation containing an autophagy inducer on the dogs' skin and hair conditions.

## 5. Conclusion

We demonstrated that Aquatide™ application improves skin and hair conditions resulting from underlying diseases; the results indicated a possible antioxidant-related mechanism. The present study could be helpful to understand the effects of autophagy on the skin and hair conditions in humans.

## Data availability statement

The original contributions presented in the study are included in the article/Supplementary material, further inquiries can be directed to the corresponding author.

## Ethics statement

The animal study was reviewed and approved by the Institutional Animal Care and Use Committee at Chonnam National University (CNU IACUC-YB-2019-26). Written informed consent was obtained from the owners for the participation of their animals in this study.

## Author contributions

YK, S-HL, and YS conducted the experiments and data analysis and wrote the manuscript. SJ contributed to manuscript preparation. H-JK conceived the experiments and revised the entire manuscript. All authors reviewed the manuscript.
