# Supplementary material for: Induction of autophagy improves skin and hair conditions in dogs with underlying diseases
Source: Front Vet Sci. 2023 Jan 26;10:1078259. doi: 10.3389/fvets.2023.1078259 (PMC9909349; doi:10.3389/fvets.2023.1078259)
Supplement: Supplementary file 1 [file Table_1.DOCX]

Supplementary Material

# Supplementary methods

## Western blot

For western blotting analysis, canine keratinocytes were homogenized, and total protein was extracted from the keratinocytes in the presence of a protease inhibitor cocktail (Sigma-Aldrich, St Louis, MO). Total protein concentrations were determined using the Bradford method. Protein samples (20µg) were resolved on 10% SDS-PAGE gels at 60V for 90 minutes and then transferred to polyvinylidene difluoride membrane (Amersham, Uppsala, Sweden) at 250mA for 120 minutes in accordance with the standard procedures. The membrane was then blocked with 5% v/v skim milk in a TBS-T buffer (TBS with 0.1% w/v Tween-20) for 1 hand reacted with the anti- Glucose regulated protein (GRP-78) antibody (diluted to 0.2µg/mL in TBS-T buffer) (R&D Systems, Minneapolis, MN), anti- Interleukin (IL) 4 antibody, anti-IL-13 antibody, and anti-β-actin antibody (diluted 1:2000 in TBS-T buffer) (Sigma-Aldrich) for 24 hours on a rocking platform at 4℃. The membrane was then washed 3 times with the TBS-T buffer for 15 minutes and incubated for 1 hour with TBS-T buffer containing anti-goat antibody (1:3000) (Sigma-Aldrich). The hybridized membrane was washed three times with TBS-T buffer for 15 minutes and visualized using medical X-ray film blue (AGFA) after treatment with ECL system reagents (GE Healthcare). Blots were analyzed using Image J (National Institutes of Health, Rockville, MD, USA).

## Real-Time RT-PCR

Total RNA from canine keratinocytes was extracted using an RNeasy kit (Qiagen, Valencia, CA) and cDNA was synthesized from 1 µg aliquots of these preparations using a WizScript cDNA synthesis Kit (Wizbiosolutions, Korea). qPCR was performed on an ABI 7900 system (Applied Biosystems, Piscataway, NJ). The expression level of each gene was normalized to that of glyceraldehyde 3-phosphate dehydrogenase (GAPDH).
